# “Art as Experience” revisited: Dewey’s aesthetic cognition theory in the age of fragmented perception

**DOI:** 10.3389/fpsyg.2026.1805282

**Published:** 2026-07-24

**Authors:** Fangting Zhang

**Affiliations:** 1 School of Arts, Nanjing University, Nanjing, China; 2 School of Physical Education and Humanities, Nanjing Sport Institute, Nanjing, China

**Keywords:** aesthetic cognition, Art as Experience, art education, fragmentation, John Dewey

## Abstract

The technological environment of the information age is reshaping our ways of perceiving art, leading to the prevalence of cognitive fragmentation and experience disconnection as a common symptom of modernity. This phenomenon is manifested in the temporariness of perception, the discontinuity of experience, the floating nature of meaning, and the lack of depth. It has triggered contemporary anxiety about whether deep aesthetic experience is possible. This paper uses John Dewey’s theory of “Art as Experience” as the core analytical framework. And a critical diagnosis of the fragmentation phenomenon is conducted through a systematic explanation of its principles of continuity, embodied cognition, consummatory experience. The study reveals that fragmentation essentially causes an incomplete state of experience and interrupts the dynamic growth process of “an experience” emphasized by Dewey. However, Dewey’s theory not only provides a critical lens but also points to a constructive reinterpretation path. This paper argues that traditional art organically promotes active integration through its inherent unity of form and context. However, digital fragmentation disrupts this natural process, requiring the aesthetic subject to make more conscious and resistant active efforts. Fragments can serve as enticers of experience to stimulate in-depth exploration, and the re-creation of digital context and the generation of personalized experience narrative become contemporary practices for reconstructing “consummatory experience”. This paper constructs a practical path of art education grounded in Deweyan aesthetics, aiming to reshape a more complete, profound and generative model of artistic cognition. Dewey’s theory of aesthetic cognition enlightens us that art education in the information age should shift toward cultivating the ability to integrate experiences, and the true “aesthetic experience” may precisely manifest as a conscious resistance to fragmentation and the reconstruction of experiential continuity, thereby providing valuable philosophical guidance for anchoring experience in a fluid era.

## 1 Introduction

John Dewey (1859–1952) was a highly prominent American philosopher, psychologist, and educational reformer of the 19th and 20th centuries. He attained global recognition through his work on democracy and education and is credited as one of the principal founders of pragmatism. And Dewey’s theory of experience remains a cornerstone of art education today, elevating art as the essential means through which lived experience is integrated and holistic growth is fostered in a democratic society (Schulz, 2025).

In art education, cognition is largely accepted as a facet of the conscious and unconscious mind (Tavin, 2010). In 1974, Brian Allison proposed a more cerebral approach to art education in his thesis *Intellectual factors in art education*, and the role of cognition in art education has been deliberated. To explore the connections formed between art, education and cognition, it is necessary to conceptualize the concept of cognition and apply it as a single and connected entity. Cognition could be defined as: knowledge, process, interdisciplinary experience and embodiment, offering a framework for understanding the meaning of cognition (Heaton, 2021).

One view holds that cognition is within the framework of knowledge. Cognitive knowledge concerns information networks that are acquired through experiences (Barrett, 1995). An artist teacher research participant rationalized cognitive knowledge by expressing: “the cog, in cognition, makes me think of the process to make something work – like the cogs in a clock, but in our brain” (Heaton, 2021).

Some scholars define cognition as a mental process (Efland, 2002). In art education, the mind engages in cognitive activities the mind engages in cognitive activities that integrate various art practices to construct knowledge. The cognitive processes in art education can be regarded as perceptual, meticulous, experiential, emotional, and bodily cognitive activities (Ojala, 2013). The cognitive processes are also regarded as the products of connectionist, existing or being present in thinking, language, and/or the experiences of art education (Hardy, 1997).

Cognition also manifested as interdisciplinarity (Song, 2012). The interdisciplinarity nature refers to the way in which cognitive connections are formed in art education. Sometimes it is cross-cultural, achieved through collaboration, through pedagogy, practice or by connecting art with other disciplines such as science (Güler, 2017). Interdisciplinary cognitive connections in art education need to be realized through visualization and recognition, as they link the individual, profession, society and culture together (Burnard et al., 2016).

Cognition in art education is also regarded as embodiment, serving as a means to help people understand themselves and the relationships between their minds, bodies and experiences (Ash, 2019; Heaton, 2021). Payne describes the practice of artist teacher as a continual process of mutual growth between the artist and the community (Payne, 2020). In this process, one learns and unlearns. To achieve growth, one is most likely to gain cognitive insights through their own experiences.

Art education is a process of cognitive exploration in which both perceptual and emotional processes also play a role. Within these relationships, cognitive connections were formed, helping to develop knowledge about cognition through the experience of art education. In Dewey’s theory of cognition, he defined education as “that reconstruction or reorganization of experience which adds to the meaning of experience, and which increases ability to direct the course of subsequent experience” (Dewey, 1916). Dewey believed that experience is a continuous interaction process between individuals and their environment by taking experience as the foundation of his philosophical system (Schulz, 2025). That means, Dewey believed that experience is a prerequisite for cognition. Experience derives from both education and the interaction between individuals and their external environment. With the progression of time, changes in how information is acquired have led to corresponding transformations in cognitive processes.

With the progress of digital era, the development of Internet technology and digital devices are leading to a revolutionary change in information acquirement and learning. The traditional methods of information acquisition are limited by geographical location and time. However, the abundant resources and flexible access methods of online media have provided convenience for fragmented information acquisition. Video-sharing platforms such as Tiktok and Bilibili support personalized and fragmented learning with their rich educational resources, broadening the breadth and depth of learning (Zhong and Xu, 2025). The online fragmented learning, which uses mobile devices as carriers, is more flexible in terms of networked learning content than traditional classroom-based learning (Zhang et al., 2015). Correspondingly, Aldholay et al. believes that online fragmented learning allows learners to access learning websites at anytime and from anywhere, which helps learners make better use of their fragmented time (Aldholay et al., 2018). However, Tang et al. believe that online fragmented learning is still in its initial stage and require further improvement, although it is feasible (Tang and Li, 2021).

The concept of “fragmentation” appeared in research on reading behavior in the 1980s, and it has been mainly applied in education and other fields (Li and Yang, 2023). Fragmented learning is an emerging learning model, which has attracted significant attention from academics and researchers due to its potential to enhance the learning outcomes of students and academic performance. However, scholars have yet to reach a consensus on the definition of fragmented learning. Hug proposed that fragmented learning refers to small-scale learning units and short-term learning activities provided through mobile devices, with a focus on imparting immediate, skill-based knowledge (Hug et al., 2006). Wang expounds the connotation of fragmented learning from two dimensions: broad and narrow. The broad sense of fragmented learning encompasses formal learning and informal learning, during which learners make use of fragmented time, resources and media. While the narrow sense of fragmented learning specifically refers to the informal learning category included in the broader sense of fragmented learning (Wang, 2016). Yang et al. point out that fragmented learning is an informal learning method. This approach partitions learning time and content, thereby enabling learners to make use of fragmented time to acquire fragmented knowledge (Yang et al., 2022). Online fragmented learning entirely depends on the interests, goals, thinking, and other needs of the learners. Accordingly, the characteristics of fragmented information acquirement include the flexibility in time, detailed content, and the portability of media, especially emphasizing the fragmentation of learning content and the miniaturization of learning tools (Zhong and Xu, 2025).

The fragmented learning brings multiple challenges to aesthetic cognition. On one hand, it may reduce the depth of the public understanding of classic art. Empirical studies in cognitive psychology have demonstrated that viewers exposed to fragmented art content consistently focus on the parts, highlights, or “essentials” of a work rather than its inherent unified form, organic structure, and subtle tension of details (Li et al., 2025; Foulsham and Cohn, 2021), thus simplifying the cultural connotations and historical context of art into isolated “symbols” or “image fragments”. On the other hand, media psychology research has confirmed that the visual impact and technical display effects frequently take precedence over the narrative content and aesthetic value in digital environments (Itti and Koch, 2001; Museum of Modern Art Australia, 2025), leading to a deviation of aesthetic cognition from its humanistic core. As Claire Bishop pointed out in *Disordered Attention: How We Look at Art and Performance Today* (Bishop, 2024), the contemporary technological environment has reshaped our viewing experience, and “disorder of attention” has become a prominent feature of this era. Art should serve as a bridge connecting individual experiences and collective culture, prompting people to reflect on life and understand human nature through profound experiences. However, it may significantly lose its critical, integrating, and enlightening power for most casual audiences when art is reduced to quick-consumable information fragments. Importantly, this does not imply an inevitable technological determinism. Recent empirical work in cognitive psychology suggests that active aesthetic subjects with prior art literacy can consciously synthesize scattered digital fragments into a coherent and meaningful complete experience post-consumption (Bara et al., 2026), and may even generate novel interpretive perspectives through the recombination of heterogeneous fragments (Gross and Schooler, 2026). Nevertheless, such active synthesis requires substantial cognitive effort, sustained attention, and foundational art knowledge, which remains inaccessible to the majority of general viewers in the current fast-paced digital media environment.

**FIGURE 1:**
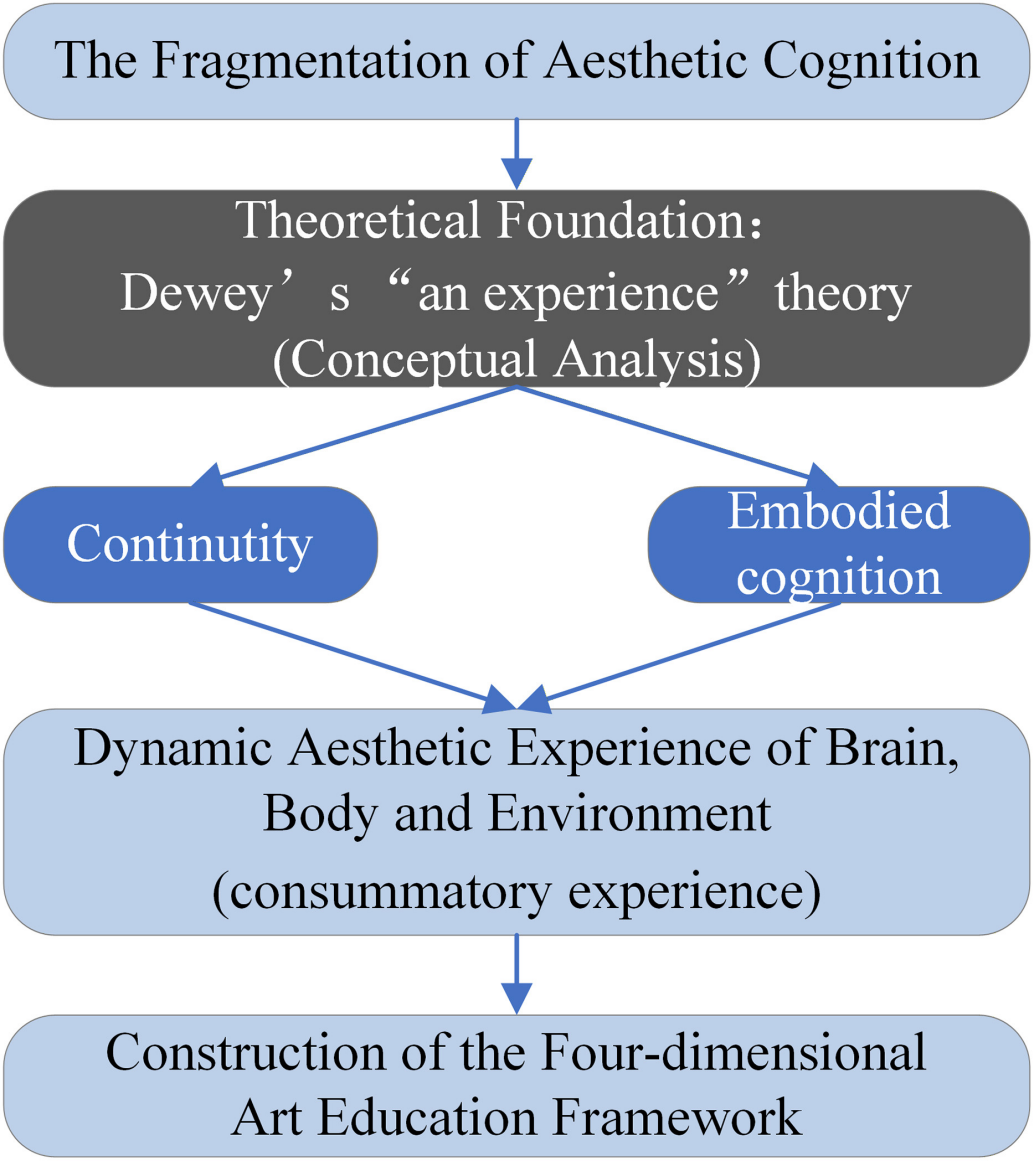
Research framework of this study.

In the context of the era of fragmented information acquisition, the formation of aesthetic cognition has undergone a transformation. How to conduct a critical diagnosis of this phenomenon using Dewey’s cognitive theory is the focus of this study. Fragmentation essentially leads to an “incomplete” state of experience and interrupts the dynamic growth process of “an experience” emphasized by Dewey. This paper argues that traditional art organically promotes active integration through its inherent unity of form and context. However, digital fragmentation disrupts this natural process, requiring the aesthetic subject to make more conscious and resistant active efforts. In this fragmented perceptual environment, the core task of the aesthetic subject shifts from engaging with the pre-existing integrated whole of the artwork to actively reconstructing experiential continuity from dispersed fragments. Fragments can serve as “enticers of experience” to stimulate in-depth exploration, while the recreation of “digital context” and the generation of “personalized experience narrative” become contemporary practices for reconstructing “consummatory experience”. An in-depth understanding of how aesthetic cognition is transformed in fragmented perception allows this study to enhance the aesthetic experience for educators in art education, thereby laying the foundation for the rebuilding experimental continuity in the digital age. The research framework of this study is schematically shown in Figure 1.

## 2 Methodology

This study adopts conceptual analysis, a core methodology in the analytic philosophical tradition, as its primary research method. The central goal of conceptual analysis is to systematically clarify ambiguous philosophical concepts, reveal their internal logical structures, and examine their applicability in new contexts (Beaney, 2014). Conceptual analysis differs from empirical research methods in that it does not depend on empirical data, and it establishes clear standards for the cross-contextual application of theories through rigorous textual interpretation and logical reasoning.

The analytical process of this study is divided into three phases. First, semantic clarification of original concepts. This study systematically examines all usages of the five core concepts through a close reading of Dewey’s works – “an experience”, “continuity”, “consummation”, “body-mind”, “sensory-motor co-ordination” – distinguishes their core meanings from peripheral meanings, clarifies the logical relationships among them, and further explores the contemporary extension of these concepts. This study extracts the core logic of Dewey’s aesthetic concepts that is independent of specific historical conditions and translates it into theoretical language applicable to digital learning environments by comparing the fundamental differences between the industrial age context in which Dewey’s aesthetic theory emerged and the contemporary digital information age context. Third, the construction of application criteria. This study formulates the necessary and sufficient conditions, grounded in the analyses from the two phases, for judging whether a given feature of a digital learning platform adheres to Deweyan aesthetic principles.

The method provides a clear, traceable, and refutable reasoning framework for the contemporary application of classical philosophical theories, avoiding subjective arbitrary analogies and leapfrog arguments. In this paper, it allows the study to systematically translate Dewey’s concepts of continuity, embodiment, and wholeness into diagnostic and constructive tools for analyzing and redesigning both traditional and digital aesthetic experiences, thereby directly supporting the core argument of this paper that resisting fragmentation and reconstructing experience continuity constitute the essence of aesthetic experience in the information age.

## 3 Cognitive transformation in aesthetic experience

The fragmented perception process characteristic of the information age pervades daily life. This “incomplete” state of experience has become the mainstream way for people to acquire knowledge. In such an era, how to critically examine this issue deserves in-depth reflection and exploration.

The core idea of Dewey’s “an experience” lies in replacing the traditional philosophical dichotomy of subject and object with “experience” to establish the natural foundation of cognitive activities. In “Art as Experience”, Dewey begins by criticizing the separation between art and life experience in modern society, arguing that this separation leads to the fragmentation of cognition (Dewey, 1987). On this basis, Dewey constructed a complete philosophical system to address this cognitive transformation task. He revealed the intrinsic attribute that aesthetic experience stems from daily experience by redefining art as the perfect state of “an experience”, thereby philosophically reconstructing the continuity between art and life. He believes that only when a large number of fragmented perceptions in daily experience (such as interrupted conversations and unfinished work) develop into “an experience” with internal integration can cognitive completeness be achieved. Specifically, he achieved it through the following key steps and they are discussed in the following sections.

### 3.1 The continuous reconstruction of aesthetic cognition

Dewey’s naturalistic philosophy of art emphasizes the differentiated continuity that runs through the entire nature. This feature is fully reflected in Dewey’s exposition of aesthetic experience: in “Art as Experience”, Dewey proposed that “This task is to restore continuity between the refined and intensified forms of experience that are works of art and the everyday events, doings, and sufferings that are universally recognized to constitute experience.” (Dewey, 1987), and the “continuity of experience” is the fundamental task of art philosophy.

First, cognitive dualism has been shattered. Dualism divides the world into two opposing domains and always assigns a higher value to one. Taking “the live creature” as his foundation, and rooted in the philosophies of empirical naturalism and instrumentalism, Dewey reinterpreted dualism not as essentially opposing existences, but as different stages or different emphases within a continuous empirical process. He argued that aesthetic experience, rather than being a mysterious ability unique to humans, evolved from the rhythmic interaction between organisms and their environment in the service of survival. Cognition, perception and emotion are all tools for a living being as a whole to adapt to the environment. Therefore, artistic activities are those in which the entire organism (including the body and mind) is fully engaged, rather than purely spiritual products. The pleasure of appreciating a painting and the satisfaction of savoring delicious food or sweating during exercise are continuous on a biological basis.

Secondly, Dewey proposed “an experience” and established the continuity of experience by redefining “experience”. Dewey distinguished between two types of experience: one is conventional experience, the fragmented, mechanical and unfinished matters in daily life. For instance, we commute numbly every day, browse information in a fragmented way and complete work mechanically. This experience is a manifestation of “fragmented perception”. The other one is “an experience”, which is an experiential process with inherent unity, integrity and emotional traits. It has a beginning, development, climax and ending, and is a self-sufficient whole. For instance, a thorough conversation, successfully solving a difficult problem, or carefully preparing and enjoying a dinner. Dewey believed that the essence of an artwork is the concentrated, intensified and elevated presentation of “an experience”. “An experience” has a complete process that starts from its own impulse, goes through a series of orderly developments, encounters resistance, forms tension, and ultimately reaches a perfect realization. It forms a sharp contrast with the fragmentation, disorder and superficiality of fragmented experiences. Practicing Dewey’s ideas means that we consciously pursue deep concentration (the state of flow) in our work and life, complete meaningful task units, and reflect on and enjoy the results, thereby restoring the continuity and depth of our thinking. In Dewey’s view, the concept of “stream of consciousness” embodies a naturalist perspective. It analyzes psychological events within the framework of the continuous interaction between the organism and the environment, thereby advocating the use of a biological behavioral description framework for psychological phenomena.

Thirdly, Dewey set out to restore the continuity between art and non-art based on the continuity of experience, developing “an experience” into “aesthetic experience”. The restoration of the continuity between art and non-art as Dewey claimed includes several levels: The first is the continuity between the experience of artworks and the experience of daily life. Experience is not limited to our encounters with artworks, and it pervades every aspect of our daily lives. The second level is to seek the continuity between high art and popular art. Further, Dewey attempts to establish a continuity between the art of beauty and the art of practicality or technology. The handicraft workshops in the Middle Ages, gave birth to modern manufacturing on the one hand, they also gave birth to the art of beauty on the other hand. The traditional view, shaped by the notion of disinterestedness in aesthetics, considers only those products not manufactured for utilitarian purposes to be works of art. Dewey believed that whether something is practical or not is not a sign to distinguish whether it is art or not. He pointed out, “The fetches of the Negro sculptor were taken to be useful in the highest degree to his tribal group, more so even than spears and clothing. But now they are fine art, serving in the twentieth century to inspire renovations in arts that had grown conventional. But they are fine art only because the anonymous artist lived and experienced so fully during the process of pro-duction” (Dewey, 1987). The artistic standards he proposed seem quite novel to us today. He proposed that “as long as there are conditions to prevent production behavior from becoming an experience in which the entire organism is alive and has its own life through enjoyment, then the product will lack beauty. No matter how useful it is for special and limited purposes, it will ultimately not make a direct and free contribution to expanding and enriching life” (Dewey, 1987). Collectively, these discussions laid the theoretical groundwork for his conception of returning art to daily life. On this basis, Dewey bridged the gap between aesthetic experience and daily life experience, and restored the continuity between the two.

### 3.2 The embodied turn in aesthetic cognition

The fragmented perception of aesthetic cognition in the information age is essentially a modernity symptom of physical and mental separation and experience fragmentation. Dewey’s thoughts on aesthetic cognition, especially his emphasis on the embodied dimension, provide profound theoretical resources and practical paths for addressing this challenge. In Dewey’s view, art is not a passive object of appreciation but an embodied experience of the interaction between the organism and the environment. This core viewpoint directly points out the root cause of the fragmentation of aesthetic cognition and provides a systematic solution.

In “Art as Experience”, Dewey systematically expounded his embodied art view. He believes that true aesthetic experience stems from the interactive process between living organisms and their environment, and it is a complete and unified experience that the subject acquires through physical participation in the world. Aesthetic cognition is not an abstract process that occurs in the “mind”, but an embodied practice realized through the participation of the body’s senses, movements and emotions. This view challenges the absence of the body and the one-sidedness of the senses in fragmented perception.

First, the body is the foundation and starting point of mental activities such as cognition, thinking and aesthetics. The mind exists within the body and its interaction with nature, which aligns with and anticipates Merleau-Ponty’s core phenomenological view that “The body is our general medium for having a world” (Maurice Merleau-Ponty, 2012).

While Dewey develops his embodied perspective from the pragmatist tradition of biological adaptation and practical experience, Merleau-Ponty’s phenomenology provides a complementary cognitive psychological foundation by elaborating how the body actively structures perception prior to conscious thought. The body schema is central to his account – a pre-reflective, dynamic system of bodily capacities and postural awareness that allows for unmediated interaction with the world. Merleau-Ponty further argues that all perception is inherently a pre-reflective bodily synthesis. Rather than assembling isolated sensory inputs into a coherent world in the mind, the body – as a unified whole – already organizes sensations into meaningful experiences before any explicit reflection. This insight directly reinforces Dewey’s rejection of Cartesian mind-body dualism and his core premise that mental life is fundamentally continuous with physical action.

Dewey’s concept of the interdependence and interaction between the body and the mind can be further understood from two aspects: on the one hand, cognition and thinking depend on the body and its activities in life. On the other hand, physical movement and activities also contain consciousness and thinking. Dewey convinced that our spiritual and mental life is deeply rooted in our physical experiences and behaviors, and the will, regarded as often considered transcendent, is essentially materialized as well, belonging to reflective bodily consciousness. “Every thought and meaning has its substratum in some organic act of absorption or elimination of seeking, or turning away from, of destroying or caring for, of signaling or responding. It roots in some definite act of biological behavior” (Dewey, 1981). Psychological actions originate from physical actions. Conversely, the body in activity and experience is not a walking corpse, and it is perceptual and conscious. Perception and sensation are not merely a matter of physical contact or visual spatial entry. “There is something present in organic action which acts as a surrogate for the remote things signified. The words make immediate sense as well as have signification. This something now present is not just the activity of the laryngeal and vocal apparatus” (Dewey, 1981). Accordingly, this fragmented perception leads not only to attentional dispersion, but also to the flattening of lived experience and the impoverishment of meaning-making. Therefore, the true emotional impact and ideological depth of art become impossible when the body is excluded from the cognitive process and experiences are reduced to unrelated fragments.

Secondly, Dewey’s embodied cognition theory is particularly prominent in art: the tools used by artists (such as brushes and chisels) are extensions of human organs. “Hands and feet, apparatus and appliances of all kinds are as much a part of it as changes in the brain. Since these physical operations (including the cerebral events) and equipment are part of thinking, thinking is mental” (Dewey, 2004), connecting bodily sensations, perceptions, and the objects in hand with thoughts. In artistic creation, the body serves not only to express emotion but also to integrate consciousness with the environment. Artists use images as the artistic medium to conceive their works, in which both thoughts are combined with materials. Our hands and surroundings also constitute the possibilities of our actions and thoughts, all of which form the very meaning and substance of how an individual embodies their mind in action through this ready-to-hand mode of being. This concept of “ready-to-hand” thinking and intervening in things is inherently consistent with Polanyi’s relevant discussions of focal awareness and auxiliary awareness. The tacit knowledge acquired through body awareness is an experience that integrates the body’s mechanisms, while the body and its surrounding environment also constitute part of perception. The perception subject and the object are not separated. The perception subject does not grasp and understand the object like a bystander or an outsider. Perception is an interactive process, and the perception subject and the object form a continuum. We acquire knowledge and meaning through physical actions and experiences, and these are then absorbed and become part of our body. Consequently, the body itself gains creativity through such active engagement.

### 3.3 Dynamic aesthetic experience of brain, body and environment

As mentioned above, Dewey pointed out that there is a profound separation between art and life, and regarded bridging this gap and restoring the continuity between the two as the fundamental task of art philosophy (Dewey, 1987). This declaration not only challenges the tradition since Kant that regards aesthetics as pure contemplation, thereby bridging the gap between art and life experience in modern society, but also reveals a dynamic cognitive essence: art is not a sacred realm isolated from life, but rather a perfect experience generated through the interaction between the organism (brain and body) and the environment. Therefore, the object presents the strongest aesthetic quality in art when sensory materials and action materials achieve the most complete mutual penetration and integration. The formation of aesthetic experience is not a simple linear composition. It presents an experience process of continuous reconstruction of the brain, body and environment through the generation of artistic perception and artistic actions. Each dimension is not an isolated existence, and they jointly shape aesthetic experience in continuous interaction. Therefore, how can aesthetic experience be strengthened and improved through the collaboration of multiple systems?

First of all, Dewey pointed out that artistic activities can “carry to new and unprecedented heights that unity of sense and impulse, of brain and eye and ear, that is exemplified in animal life, saturating it with the conscious meanings derived from communication and deliberate expression” (Dewey, 1987). This combination is not a static balance but a dynamic synergy – when artists are confronted with creative challenges, different regions of the brain are activated and new connection patterns are formed, which is precisely the neural basis of artistic creativity. Dewey keenly perceived that artistic creation requires the integrated participation of the entire brain. This view is supported by modern neuroscience: The right brain is mainly responsible for processing visual-spatial information, emotional expression and intuitive thinking. In artistic creation, the right brain enables us to perceive the overall form and emotional atmosphere. The left brain mainly processes language, logic and analytical thinking. In artistic creation, the left brain participates in planning the creative process and analyzing the form and structure.

Secondly, the body serves as a cognitive channel for sensory movements. Dewey regarded the body as the core medium of aesthetic cognition, a view that completely overturned the traditional aesthetic tendency to marginalize the body. Dewey believed that “Every experience, of slight or tremendous import, begins with an impulsion, rather as an impulsion” (Dewey, 1987), and the original driving force of artistic creation is precisely this physical impulse – a longing for expression and a desire to create. The impulse is transformed into creative expression through the integration of multi-channel perception. In Dewey’s theory, the body is not only a receiver of sensations but also a creator of meaning. He held a biologically grounded view that we should not only use our bodies to experience and feel but also use our hearts to experience on the basis of physical sensations when facing life experiences. This is what Dewey called “fuse with qualities of senses that go below the surface” (Dewey, 1987). This integration endows visual art with a sense of music and gives music the characteristics of a picture, forming a synesthetic experience of art. In artistic creation, every arm movement (movement system) made by a painter when mixing colors alters the color relationship on the canvas (environment), and the perception of color changes by the eyes (sensory system) triggers the brain to adjust the next action (brain), thus forming a creative cycle. Children can gain more aesthetic pleasure from it than adults, because adults have been bound by a “practical attitude”, while children maintain a free coupling of physical movement and perceptual exploration.

Emotion can serve as a continuous bond: In Dewey’s theory, emotion is not a psychological state independent of cognition, but an integrating force that “constitutes the unity of experience”. When dancers improvise, their body movements (motor systems) are driven by the rhythm of the music (auditory input), while emotional energy runs through the movement sequence, allowing the improvisational segments to form an overall experience with internal logic. Dewey opposed regarding emotion as “what is expressed”, advocating that emotion arises from “press itself out in” – a dancer’s emotion stems not only from muscle tension (the body) but also from the musical atmosphere (the environment), and is the product of multi-factor interaction rather than a priori existence. For instance, thousand-Hand Guanyin, a renowned Chinese dance performance, exemplifies this integrative principle. Its profound impact stems from the seamless fusion of the dancers’ precise choreography (motor system), the reflective glow of golden costumes (visual environment), and the resonant Buddhist music (auditory input), all integrated by the underlying emotion of compassion. These elements coalesce into an inseparable whole, evoking a visceral tremor within the bodies of the audience.

Thirdly, art is formed as “an experience” through the high-level integration of the brain, eyes and ears. The true formation from “an experience” to an aesthetic experience can only be achieved through a long-term and creative interaction with the environment, thereby strengthening the contextual embedding and social interaction of aesthetic cognition. This environment encompasses both the natural environment and the social environment. On the one hand, the physical environment serves as an extension of artistic expression. The elasticity of the brush (object properties), the water absorption of Xuan paper (environmental characteristics), and the strength of the wrist (body) form a dynamic balance system when calligraphers are creating. Every pause (adjustment of movement) during the process of brushwork is not only an adaptation to the reaction between paper and ink (environmental feedback), but also relies on the brain’s memory invocation of the calligraphy program (the brain). This four-dimensional interaction (body – tool – material – mind) extends cognition beyond the body, making the environment an inseparable cognitive partner in artistic creation. On the other hand, the social environment serves as the soil for the generation of meaning. Dewey believed that art broke down the barriers that separated people and were impenetrable in their daily contacts. Aesthetic cognition is essentially a form of social cognition by sharing artworks, we transcend the limits of personal experience and enter a shared world of meaning. Art is such a form of communication that is shared with others, creates life and promotes social progress. Emphasizing communication is Dewey’s consistent position: “For communication is not announcing things, even if they are said with the emphasis of great sonority. Communication is the process of creating participation, of making common what had been isolated and singular; and part of the miracle it achieves is that, in being communicated, the conveyance of meaning gives body and definiteness to the experience of the one who utters as well as to that of those who listen” (Dewey, 1987). The meaning of the world originates from the act of communication. Communication is not only an activity that brings individual members into the community as participants rather than onlookers, but also a process of creation and sharing. The meaning is enriched, deepened and consolidated through such sharing. In this regard, we can counter the problem of superficial cognition brought about by fragmentation and enhance the overall cognition and in-depth understanding of culture by re-embedding art into its cultural and historical context and social practice network.

## 4 Reconstruction: from fragmentation to completeness

In Dewey’s theory of aesthetic cognition, aesthetic fragmentation is essentially a phenomenon of lacking bodily participation and the disintegration of holistic experience. Algorithmic recommendation systems interrupt the natural flow and continuous construction of experience, reducing aesthetic cognition to a random collage of isolated fragments.

Art consumption in the digital media environment often simplifies the body to mere “eyeballs” and “fingers”, depriving it of the possibility of multi-sensory collaborative participation. As Merleau-Ponty noted, this simplification profoundly disrupts the body schema: the pre-reflective bodily synthesis of the body as a unified perceptual system is artificially severed by digital interfaces, with vision and touch isolated from the overall sensory-motor apparatus. We no longer perceive artworks as holistic presences that engage our entire being, but merely as flat images that stimulate our retinas and fingertips. This sensory truncation is not simply a quantitative reduction in the number of senses involved, but a qualitative transformation in the very nature of perception, reducing the rich embodied encounter with art to disembodied cognitive processing of visual information.

The de-contextualization feature of the virtual environment further strips away the organic connection between art and its cultural soil and social context. Dewey’s reconstructed experiential aesthetic model guides the audience from passive and discrete information receivers to active and integrated constructors of artistic experience through “continuous reconstruction”, “the embodied turn” and the “brain-body-environment” in aesthetic cognition. It provides core theoretical resources and practical paths for solving the dilemma of fragmentation, which can be expanded from the following four dimensions:

First, create “continuous” art projects to weave consummatory experience in a diachronic context. The teaching reform strategies proposed in this study are primarily designed for public art education (general education courses) in Chinese universities and colleges. This group is particularly affected by the problem of fragmented cognition in the digital age: most students lack systematic artistic training, and their artistic cognition mainly comes from fragmented images and short videos on social media, making it difficult to form a complete aesthetic experience system. Therefore, reconstructing the art learning process based on Dewey’s philosophy of experience has especially important practical significance for improving the effectiveness of public art education. The primary feature of fragmented cognition is the breakage and interruption of experience. Dewey’s “continuity” principle states that experience is a fluid whole. Current experience stems from past experience and leads to future experience, thus forming a continuum of meaning growth. Therefore, art education should abandon the fragmented imparting of knowledge and skills training, and replace them with “project-based courses” that have inherent logical connections. In this way, cross-class and cross-disciplinary Project-Based Learning (PBL) is promoted, completely discarding the “knowledge point” indoctrination model of getting to know an artist and appreciating a piece of work in one class. Instead, core art projects that last for several weeks or even the entire semester are designed. For instance, in a project themed “Memories of the Community”, the work can be structured into three phases: Phase One (Investigation and Perception): Students enter the community, take photos with their mobile phones, record with sketchbooks, and interview residents (connecting life experiences and the past); Phase Two (Interpretation and Transformation): Research relevant artists and learn the corresponding artistic techniques (now); Phase Three (Creation and Presentation): The group collaborates to create a mixed-media installation or a set of narrative paintings, and holds an exhibition at the community center to explain the concept of the work (realization and feedback, leading to the future) to the residents. This process simulates the complete course of “an experience”: with a beginning, development, climax and ending, integrating all discrete “knowledge points” (artists, techniques, history) into a purposeful and emotionally engaged continuous narrative. Alternatively, around a specific theme (such as “ecological care”), cross-cycle and multi-stage exploration and practice can be carried out, covering a consummatory experience process including perception and observation, material experimentation, creative expression and reflective display. This approach helps students build an organically integrated cognitive structure by fostering meaningful connections between new and old experiences, thereby overcoming the discontinuity and superficiality of fragmented perception.

In the digital environment, highly continuous artistic projects can also be designed – projects that even break through the temporal and spatial limitations of physical artworks, building an open and continuously growing experience continuum. For example, an interactive digital narrative map project can be designed with the same theme of “Community’s Memories”:

Stage 1 (Investigation and Perception): Students’ photos taken with mobile phones, recorded interview audio and video, are no longer just materials for the physical exhibition, but are directly uploaded to a digital platform based on Geographic Information System (GIS), marked at the corresponding positions on the community map.

Stage 2 (Interpretation and Transformation): Students use tools such as Figma and Jing to process the original materials into various forms of digital narrative works, such as text and images, short videos, podcasts, etc., and establish hyperlinks between different memories on the platform, forming a network of interrelated memories.

Stage 3 (Creation and Presentation): The final outcome is not a one-time physical exhibition, but an online digital map that can be continuously updated. Community residents can scan the QR code to access, view the students’ works, and can also register an account to upload their family photos, stories of old objects and life memories, and become co-creators of the project.

Stage 4 (Extension and Growth): This digital map can be continuously operated as a long-term project of the class. Students of different years can continue to add content on the original basis, allowing the community’s memories to continuously grow and persist in the digital space.

This digital project perfectly embodies Dewey’s principle that Experience is a continuously growing continuum. It breaks the limitation of physical projects where “the exhibition ends and the project ends”, allowing experience to extend from the past to the present and continuously lead to the future. At the same time, the hyperlink structure is not a fragmented random collage, but an organic whole constructed by students and community residents with inherent meaning connections.

Second, emphasize “embodied” artistic participation: activate overall perception throughout all the senses. Fragmented perception is often “de-physicalized”: it merely engages vision for rapid scanning while isolating other senses from the deep participation of the body. Dewey’s view of embodied cognition holds that thinking is not an isolated event occurring in the brain, but rather an overall activity in which the organism wholeheartedly participates to adapt to its environment. Artistic experience is deeply rooted in the body’s sensory and motor systems, and artistic perception should thus be restored to a full-body activity involving the coordination of multiple senses. Dewey emphasized that the interaction between “doing” and “receiving” is at the core of experience formation. Art education must enable students to “do” with their own hands. Not only should a variety of materials (clay, wood, fibers, digital media) be provided, but also sections should be designed to allow students to deeply explore the physical properties of materials: the moisture and plasticity of soil, the texture and toughness of wood, and the viscosity and fluidity of pigments. In the process of “struggling” and “conversing” with it, students’ cognition is carried out through the muscles and sensations of their fingertips, arms, and even the entire body. For instance, in teaching color, we should guide students not only to use their eyes but also to engage in outdoor sketching to perceive natural light variations, and to mix pigments themselves to grasp subtle chromatic nuances. In the sculpture class, emphasis is placed on tactile experience and physical expression to deepen the understanding of form and material. They do not “remember” a concept in their minds, but rather “understand” formal laws such as weight, balance, resistance and harmony through their bodies. This embodied knowledge is profound, holistic, and indelible. It fundamentally differs from the fragmented information about a material seen on a screen, and can effectively counter the sensory isolation brought about by digitalization. In doing so, it makes aesthetic cognition a vivid, full-bodied experience.

It is worth noting that embodied cognition is not limited to being realized through physical materials. Contemporary digital technologies have enabled multi-sensory embodied participation. For instance, students not only see three-dimensional models on the screen for digital sculpture creation, but also feel the hardness, stickiness and resistance of the virtual clay through the tactile feedback gloves, and the movements of their hands directly translate into changes in the shape of the model. Besides, students can paint in three-dimensional space using their entire bodies through VR painting tools (such as Tilt Brush), and the swinging of their arms and the turning of their bodies all become part of the creation, breaking the traditional physical limitations of flat painting. These digital tools do not deprive the body of its participation, instead, they expand the boundaries of bodily perception, allowing students to experience form, space and material in a new way. Distinct from the passive consumption of digital art on a screen, active, embodied digital creation can equally engender a deep and holistic artistic cognition.

Third, build a “contextualized” learning environment: generate meaning in dynamic interaction. Dewey’s view of cognition is ecological, emphasizing that cognition occurs in the dynamic interaction among the brain, body and environment. Fragmented artistic perception often strips works of art from their original cultural, historical and physical contexts, turning them into suspended symbols. Therefore, it is of vital importance to reconstruct the “dynamic coupling of brain, body and environment”. The art classroom should transform from a place for skills transmission to a supportive cognitive environment, integrating material workshops, exhibition areas and discussion spaces, and reasonably incorporating digital and traditional media. For instance, transform the traditional “row seating” studio into a fusion of a studio, workshop and exhibition hall. The space is divided into material, creation, discussion, and display areas, allowing students to move freely, communicate, and collaborate. Tools, materials, semi-finished products in the environment, as well as classmates’ works, all become “environmental components” that stimulate thinking. Students are no longer isolated learners but engage in continuous interaction with a rich and potentially stimulating environment, in which their cognition is dynamically shaped and transformed.

Besides, the learning field should be expanded. Art galleries, communities and natural spaces are welcome to be incorporated into the teaching environment, and students are encouraged to generate systematic cognition through continuous interaction with the rich environment. At the same time, cultural and historical contexts should be embedded in the learning field. For instance, when introducing a Gothic church, one should not merely display pictures to teach its artistic style in isolation. Students can understand the structural mechanics principles of flying buttoning walls through the assembly of architectural models (embodied). One can feel its religious atmosphere (multi-sensory) by listening to the Gregorian chant. Discussing the functions and symbolic meanings of the church through role-playing (such as an architect, a priest, or a citizen) (contextualized). In this way, art is reduced to a concentrated embodiment of the technology, beliefs and social life of an era. What students establish is a three-dimensional, networked cognitive connection, rather than an isolated style label to be memorized. This reconstruction transforms students from passive recipients into active constructors of meaning.

Digital technology can also create highly contextualized learning environments. For instance, historical art scenes can be reconstructed by using digital twin technology. Students can “enter” the Florence of the Renaissance period through VR devices, closely observe Botticelli’s “The Birth of Venus” in the virtual exhibition hall of the Uffizi Gallery, listen to the street music at that time, see the surrounding buildings and pedestrians, and even have a conversation with the virtual artist. This digital context is not a simple replication of the physical context, but can provide experiences that the physical context cannot achieve. Students can “touch” the virtual paint, observe the creation process of the painting, and even modify a certain part of the painting, experiencing the decision-making process of the artist. Moreover, a hybrid learning environment that integrates online and offline elements can be built. Students conduct experiments in physical classrooms, and upload the experimental process and results to the online learning platform at the same time, allowing cross-regional communication and collaboration with students from other schools, thereby expanding the learning context from a single classroom to a global scale.

Fourth, cultivate the habit of “reflective” thinking: integrate fragments in metacognition. Another consequence of the influx of fragmented information is the weakening of the ability to reflect. Dewey regarded “reflective thinking” as a key tool for transforming chaotic and ambiguous situations into clear ones, and it is the mental guarantee for the integration and elevation of experience. Embed a “review session” at each stage of the project, but this is not the final evaluation, rather, it is a formative reflection. Adopt the framework of “description – analysis – interpretation – evaluation” to guide students to have in-depth dialogues about their own or their peers’ works. Questions such as: “What happened when you tried to mix these two colors?” What does your body feel? (Connecting with embodied experience) “How has this element changed from your initial idea?” What led to this evolution?” (Tracing Continuity) “If your work were placed in another environment, how would its meaning change?” (Think about the situation). This kind of dialogue forces students to pause and examine their decision-making process, bringing subconscious operations to the conscious level. Fragmented actions and feelings are then integrated into a logical narrative through critical dialogue that runs throughout the entire process.

Digital platforms can serve as powerful tools for fostering reflective thinking. For instance, the entire creative process of students can be automatically recorded by leveraging the “process portfolios” feature of online learning management systems (LMS), including each draft, revision notes, discussions with teachers and classmates, as well as screen recordings and operation logs during the creation process. During the reflection stage, students can review their entire creative process, analyze the evolution of their decisions, identify the factors that influenced their choices, and pinpoint where improvements can be made. Additionally, “digital reflection tools” can be designed to guide students to follow Dewey’s reflective thinking steps and organize fragmented creative actions into a logical narrative. For example, the tool will automatically prompt students: “What problems did you encounter at this stage? How did you solve them? How did this solution affect your subsequent creation?” Through this approach, digital platforms not only record the learning outcomes but also the learning process, helping students integrate fragmented experiences into a complete whole.

## 5 Conclusion

This study addresses a universal symptom of modernity in the information age – the fragmentation of cognition and the rupture of experience. Using the concept of “an experience” from American pragmatist philosopher John Dewey’s *Art as Experience* as its core analytical framework, it systematically elaborates three central principles: continuity, embodied cognition, consummatory experience. On this basis, the study offers a critical diagnosis of the predicament of artistic reception in the digital age.

First, it reveals that the essence of fragmentation lies not merely in an increased quantity of information, but in the loss of experiential integrity. Fragmentation interrupts the dynamic growth process of “an experience” from its inception to its successful completion, ultimately resulting in the transience of perception, the floating nature of meaning, and the systematic absence of aesthetic depth.

Second, this study clarifies the fundamental differences in how traditional art and digital art integrate experience. Traditional art organically guides the aesthetic subject toward an active synthesis of experience through its inherent formal and contextual unity. In contrast, digital fragmentation disrupts this natural process, requiring the aesthetic subject to shift from passively receiving a pre-integrated artistic whole to actively reconstructing the continuity of experience from scattered perceptual fragments.

More importantly, this study moves beyond the one-sided critical perspective that views fragmentation as purely negative. It proposes that fragmentation itself can serve as “enticers of experience”. Fragmentation can be transformed through the re-creation of digital contexts and the generation of personalized experiential narratives, from a momentary perception into the starting point for constructing a complete aesthetic experience. Based on the above theoretical findings, this study has constructed a practical path for aesthetic education based on Dewey’s embodied aesthetics from four core dimensions, aiming to reshape a complete, in-depth and generative contemporary art cognition model.

The thought of Dewey’s “Art as Experience” proposed nearly a century ago, not only accurately anticipated the spiritual predicament of contemporary people in the technological era but also provides us with a foundational philosophical orientation for transcending fragmentation. Genuine aesthetic experience is not a passive adaptation to fragmentation, but rather a conscious resistance to it and an active reconstruction of experiential continuity. By cultivating students’ ability to integrate experience and construct meaning, aesthetic education can not only effectively respond to the cognitive challenges of the technological era but also return to its essential educational mission – promoting the holistic development of individuals in terms of senses, emotions, reason, and values. Ultimately, it approaches Dewey’s ideal of “Art as Experience” making art a spiritual foundation through which people can anchor themselves and connect with others and the world in this fluid era.

## Data Availability

The original contributions presented in the study are included in the article/supplementary material, further inquiries can be directed to the corresponding author.

## References

[B1] Aldholay A. H. Isaac O. Abdullah Z. Ramayah T. (2018). The role of transformational leadership as a mediating variable in DeLone and McLean information system success model: The context of online learning usage in Yemen. *Telemat. Inform.* 35 1421–1437. 10.1016/j.tele.2018.03.012

[B2] Ash A. (2019). The unfamiliar grey matter(s): Talking brains. *Res. Art. Educ.* 2 1–22. 10.54916/rae.118910

[B3] Bara I. Ramsey R. Cross E. S. (2026). Art knowledge training shapes understanding, inspires creativity and stimulates thinking. *Rl. Soc. Open. Sci.* 13:250805. 10.1098/rsos.250805

[B4] Barrett E. (1995). *Sociomedia: Multimedia, Hypermedia and the Social Construction of Knowledge*, 3rd Edn. Cambridge, MA: MIT Press.

[B5] Beaney M. (2014). “Analysis,” in *The Stanford Encyclopedia of Philosophy (Fall 2014 Edition)*, ed. Zalta E. N. (Stanford, CA: Stanford University).

[B6] Bishop C. (2024). *Disordered Attention: How We Look at Art and Performance Today.* London: Verso.

[B7] Burnard P. Dragovic T. Flutter J. Alderton J. (2016). *Transformative Doctoral Research Practices for Professionals.* Rotterdam: Sense Publishers.

[B8] Dewey J. (1916). *Democracy and Education: An Introduction to the Philosophy of Education.* New York, NY: Macmillan Publishing.

[B9] Dewey J. (1981). “The later works, 1925-1953, Volume 1: 1925,” in *Experience and Nature*, ed Boydston J. A. (Carbondale and Edwardsville: Southern Illinois University Press).

[B10] Dewey J. (1987). “The later works, 1925-1953, Volume 10: 1934,” in *Art as Experience*, ed Boydston J. A. (Carbondale and Edwardsville: Southern Illinois University Press).

[B11] Dewey J. (2004). *Essays in Experimental Logic.* Mineola, NY: Dover Publications.

[B12] Efland A. (2002). *Art and cognition.* New York, NY: Teachers College Press.

[B13] Foulsham T. Cohn N. (2021). Zooming in on visual narrative comprehension. *Mem. Cognit*. 49 451–466. 10.3758/s13421-020-01101-w 33021728

[B14] Gross M. E. Schooler J. W. (2026). Expanding minds: Artistic film promotes conceptual expansion and verbal creativity. *Psychol. Aesthet. Creat. Arts* 10.1037/aca0000852 [Epub ahead of print].

[B15] Güler A. (2017). “Exploring a/r/tography in an interdisciplinary way: Touching music in visual art practices,” in *Building Interdisciplinary and Intercultural Bridges: Where Practice Meets Research and Theory*, eds Burnard P. Ross V. Dragovic T. Powell K. Minors H. Mackinlay E. (Cambridge, MA: BIBACC Publishing), 158–165.

[B16] Hardy C. (1997). Semantic fields and meaning: A bridge between mind and matter. *World Futures* 48 161–170. 10.1080/02604027.1997.9972614

[B17] Heaton R. (2021). Cognition in art education. *Br. Educ. Res. J*. 47 1323–1339. 10.1002/berj.3728

[B18] Hug T. Lindner M. Bruck P. A. (2006). *Microlearning: Emerging Concepts, Practices and Technologies After E-Learning.* Innsbruck, AU: Innsbruck University Press.

[B19] Itti L. Koch C. (2001). Computational modelling of visual attention. *Nat. Rev. Neurosci*. 2 194–203. 10.1038/35058500 11256080

[B20] Li H. Li J. Hao X. Liu W. (2025). Behavioral and eye-tracking investigation of event segmentation following short video watching. *NPJ Sci. Learn*. 10:86. 10.1038/s41539-025-00378-3 41298532 PMC12657513

[B21] Li Z. Yang Y. (2023). Determinants of college students’ online fragmented learning effect: An analysis of teaching courses on scientific research software on the Bilibili platform. *Sustainability* 15:16023. 10.3390/su152216023

[B22] Merleau-Ponty M. (2012). *Phenomenology of Perception.* New York, NY: Routledge.

[B23] Museum of Modern Art Australia. (2025). *Mona Lisa vs. the Algorithm: Why the World’s Most Famous Painting Would Fail on Instagram.* Available online at: https://momaa.org/mona-lisa-vs-the-algorithm-why-the-worlds-most-famous-painting-would-fail-on-instagram/ (accessed May 21, 2026).

[B24] Ojala M. (2013). Constructing knowledge through perceptual processes in making craft-art. *Techne. Ser. Res. Sloyd Educ. Craft Sci. A* 20 62–75.

[B25] Payne R. (2020). The shock of the new. *Int. Art Design Educ*. 39 724–738. 10.1111/jade.12317

[B26] Schulz T. S. (2025). The concept of reflection in the work of John Dewey. *J. Philos. Educ.* qhaf073. 10.1093/jopedu/qhaf073 [Epub ahead of print].

[B27] Song Y. (2012). Educating for peace: A case study of a constructivist approach to understanding peace through artistic expression. *Creat. Educ.* 3 79–83. 10.4236/ce.2012.31013

[B28] Tang J. Li J. (2021). Feasibility investigation and development exploration on popularizing the method of English fragmented mobile learning of undergraduates. *J. Lit. Art Stud*. 11 504–508. 10.17265/2159-5836/2021.07.008

[B29] Tavin K. (2010). Six acts of mis-cognition: Implications for art education. *Stud. Art Educ.* 52 55–68. 10.1080/00393541.2010.11518823

[B30] Wang Z. (2016). Fragmentation of learning and its countermeasures in mobile internet era: From “fixed deposit by installments” learning strategies to “internet+” classroom. *J. Distance Educ*. 4 9–16.

[B31] Yang Y. Zhang Y. Song R. (2022). Analysis of fragmented learning of college students under the background of online education. *J. Adv. Educ. Philos*. 6 45–50. 10.36348/jaep.2022.v06i02.001

[B32] Zhang K. Li Y. Yang X. (2015). The research of cognitive disorder in network fragmented learning. *Mod. Educ. Technol.* 2 88–94.

[B33] Zhong H. Y. Xu J. B. (2025). The effect of fragmented learning ability on college students’ online learning satisfaction: Exploring the mediating role of affective, behavioral, and cognitive engagement. *Interact. Learn. Environ*. 33 5805–5819. 10.1080/10494820.2025.2488142

